# Everybody's Page

**Published:** 1893-02-04

**Authors:** 


### Everybody's Page.

Why is it, asks a weekly contemporary, that typhoid fever seems to have taken so firm a hold on the very class of persons in England whose surroundings would most naturally seem to exempt them from the fell sway of illnesses resulting from insanitary causes? And yet the sick-room reports of our nobility are certainly two-thirds of them cases of typhoid fever, but how to account for this, in these days of ultra-sanitary reform, is a riddle beyond the power of our answering.

Frog-farming is being introduced into America with great success. On the banks of the Mississipi a tract of land of several hundred acres is already entirely devoted to the cultivation of these new victims to the epicurean palate. The farming of these frogs appears to be a lucrative business, there being a demand for many thousands each morning, and the outlay for their keep substracting little from the yearly income. In the instance of the farm on the Mississipi, quoted above, the American breed has been much improved through a consignment from France of a much larger size. These froggeries appear to be a very ingenious method of turning marshes and low-lying ground to an excellent account.

It is indeed turning the tables when our French neighbours commence an attack on the sanitation of our habitations. A recent number of a French journal has a long article exposing the unwholesome arrangement of the Highland Crofters' dwellings. We rather think ourselves that the attack has been based upon a series of similar articles which appeared in a British newspaper. Many of the assertions put forward therein were indignantly denied by those who were well acquainted with the lives and customs of the Crofters. Despite what has been said, the Crofters are, on the whole, a hardy, healthy race, and the habits which are said to prevail amongst them are no more insanitary than those of the majority of dwellers in the narrow streets in French towns where plenty of air and sea breezes do not help to mitigate the evils of unhealthy habitations and customs.

A new theory in anæsthetics is reported from Vienna. Dr. Schleich, the discoverer, secures absolute immunity from pain by local action only, the consciousness of the patient being maintained. The process, a subcutaneous injection of a solution of salt or sugar, or of plain distilled water even, appears most simple. It offers none of the objections to be raised against the use of narcotics, and numerous experiments have proved its success. The immunity from pain is obtained by temporary paralysis of the nerve caused by the action of the cold injection.

It is to be hoped that the latest measure which the Russian Prefect of Police has seen fit to enact will only be of a temporary character. The victims of this new regulation are the wretched house porters at St. Petersburgh—Dvoraks as they are called. These men are now compelled to keep watch over the establishments to which they are connected, from seven o'clock in the evening until six in the morning, during summer; and the surveillance is required to be in inverse ratio to the duration of daylight for the other seasons of the year. It is only to be conjectured that the expression of the Nihilistic opinion on this inhuman measure will assume a somewhat practical form.

It is quite difficult to realise that needles have been manufactured for so long as two centuries, so completely do they bear the stamp of the advanced mechanism of the latest civilisation. Those of English manufacture still bear the palm, both as to quality and cheapness in production. Redditch is the centre of the trade, and as many as 20,000 persons obtain employment in the factories. Although the needle of to-day is very much as the needle of yesterday, the methods by which it is produced are very much improved. Previously needle making was one of the most injurious of occupations owing to the minute particles of steel which filled the air during the grinding process, and which were swallowed by the employés. Now an air blast brought to bear upon the grindstone directs the dangerous scattering and the manufacture can be carried on with impunity.

